
JOURNAL INFORMATION
==============================
NLM Title Abbreviation: ScientificWorldJournal
Journal ID: ScientificWorldJournal
NLM Journal ID: 101131163
Journal ID: TSWJ

The Scientific World Journal

ISSN: 2356-6140
EISSN: 1537-744X
Publisher: Wiley

ARTICLE INFORMATION
==============================
PMCID: PMC5956441
PMID: 15349556
DOI: 10.1100/tsw.2004.86
Article ID: 296194
Article version: 1
Subjects: Research Article

Evaluation and Management of Male Urinary Incontinence

Stone Anthony R.
Nelson Roscoe S.
University of California, Davis, Sacramento, CA, USA
Academic Editor: Anthony Atala

Publication date: 2004
Electronic publication date: 2004 Jun 7
Volume: 4
First page: 330
Last page: 345
Copyright: Copyright © 2004 Anthony R. Stone and Roscoe S. Nelson.
Copyright year: 2004
License: This is an open access article distributed under the Creative Commons Attribution License, which permits unrestricted use, distribution, and reproduction in any medium, provided the original work is properly cited.
License URL: https://creativecommons.org/licenses/by/3.0/

==============================
Articles
*TheScientificWorldJOURNAL* (2004) 4 (S1), 330–345
ISSN 1537-744X; DOI 10.1100/tsw.2004.86

### Evaluation and Management of Male Urinary Incontinence

Anthony R. Stone, M.D. and Roscoe S. Nelson, M.D.
*University of California, Davis, Sacramento, California*

*Previously published in the Digital Urology Journal*

**DOMAIN**: urology

### INTRODUCTION

The male lower urinary tract is well adapted to the rigors of everyday life in providing an effective low-pressure storage organ and a sophisticated, relatively trouble-free sphincter mechanism. The majority of male incontinence cases encountered presently are related to prostate pathology, many being due to sphincteric weakness following radical surgery.

This review article will discuss the anatomy and neurophysiology of the lower urinary tract in the male and the causes, evaluation, and management of incontinence.

### FUNCTIONAL ANATOMIC CONCEPTS

Our knowledge of the lower urinary tract continues to evolve with the utilization of innovative neuroanalytical techniques such as immunohistochemistry and nerve-mapping studies.

The sphincteric mechanism should be considered as two distinct but functionally related parts, namely the proximal portion, or bladder neck (internal sphincter), and the distal sphincter mechanism (external sphincter, intrinsic and extrinsic portions) (Figure 1)[1]. The proximal (internal) sphincter is, for all intents and purposes, the bladder neck. It consists of smooth muscle fibers derived from those in the trigone and arranged in a circular sphincter-like fashion. Studies have shown that there is abundant alpha-adrenergic as well as cholinergic innervation.[2] Functionally, this portion of the sphincter mechanism remains closed and leak-proof at all times during bladder filling. It opens in response to detrusor contraction during the emptying phase of the micturition cycle. It will also open in response to any abnormal detrusor activity, such as detrusor instability or hyperreflexia. It is unclear whether opening of the bladder neck is due to inhibition of sympathetic influences or to parasympathetic stimulation. It has been suggested that the bladder neck's response to contraction is to open due to the arrangement of smooth muscle fibers in this area.[3]

The bladder neck also prevents retrograde ejaculation. It is unclear whether this aspect of sphincter function is produced by a separate preprostatic sphincter or the proximal sphincter itself. Evidence for the presence of a specific preprostatic ejaculatory sphincter may be gained from the clinical observation that following bladder neck incision, only 15% of patients complain of retrograde ejaculation.[4]

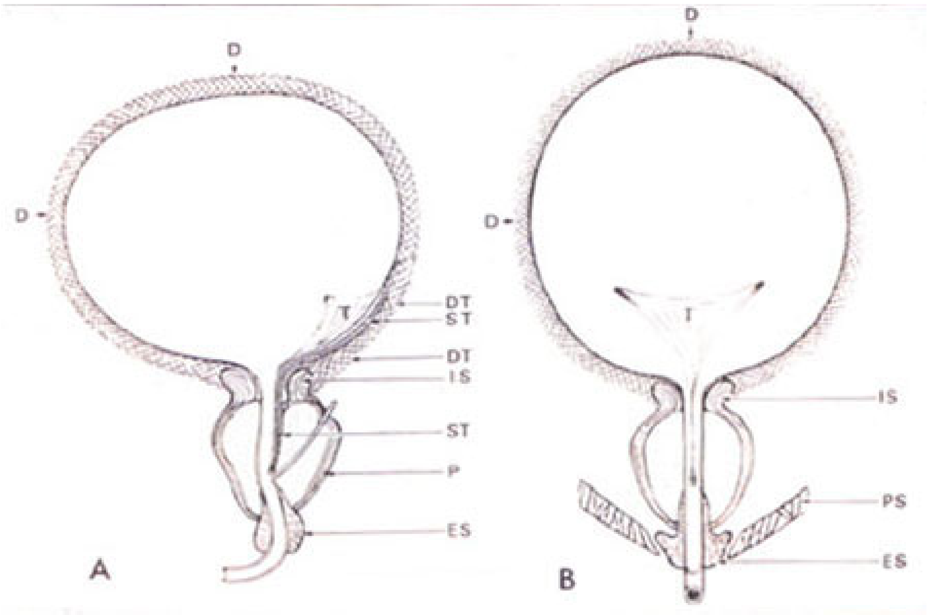

**FIGURE 1.** Gosling's interpretation of the male lower urinary tract. T, trigone; D, detrusor muscle; DT, deep trigone; ST, superficial trigone; IS, internal urethral sphincter; P, prostatic capsule; PS, external intrinsic striated urethral sphincter; PS, periurethral striated muscle. (Borrowed with permission from Urology Clinic of North America.)

The external (distal) sphincter mechanism consists of two parts, intrinsic and extrinsic. Conventionally, this sphincter has been described as a membrane or diaphragm through which the membranous urethra passes. This is, however, a gross oversimplification of this complex structure.

The intrinsic portion of the external sphincter consists of periurethral smooth muscle and specialized striated fibers. Proximally, this sphincter commences at the level of the verumontanum, but it has been shown that this margin is variable, such that functional sphincteric tissue may arise well within the prostatic urethra.[5] This portion of the sphincter consists not only of smooth muscle fibers, but also slow-twitch striated muscle, with the characteristic of a sustained fatigue-resistant contraction. This structure is sometimes referred to as the rhabdosphincter because of its striated component.[6]

The extrinsic portion of the distal sphincter is often called the urogenital diaphragm. This simplistic descriptive term implies a sheath of muscle through which the membranous urethra passes and which supports the prostate superiorly. Anatomic studies have shown that the muscle adjacent to the membranous urethra forms a ring around the urethra (puborectalis).[7] This sphincter contains normal diameter, fast-twitch striated fibers which can thus only momentarily increase urethral resistance during periods of increased intra-abdominal pressure.[8]

### INNERVATION OF THE SPHINCTER MECHANISM

It would be conceptually satisfying to adhere to a simple theory of sphincteric innervation, namely that the bladder neck is supplied with a rich plexus of sympathetic neurogenic endings, the slow-twitch fibers of the distal sphincter by branches of the pelvic plexus (parasympathetic), and the fast-twitch pelvic floor component by the pudendal nerve (somatic). Recent studies have shown that this neat functional division of innervation may not be entirely correct, with reports that the pudendal nerve may supply both divisions of the distal sphincter mechanism as well as receiving nerves derived from the pelvic plexus.[9] The innervation of the sphincter mechanism requires further clarification.

### CONTINENCE MECHANISMS IN THE NORMAL MALE

Continence depends on a balance between bladder storage and urethral resistance. During the filling phase of the micturition cycle, the bladder is able to accommodate increasing volumes of urine at pressures that always remain lower than 15 cm $H_2O$, whatever the volume. Stretch receptors in the detrusor send sensory impulses, probably via the sympathetics, that allow perception of bladder filling. Despite this increase in the volume within the bladder, the detrusor will remain inhibited until the patient wishes to void. Thus, water cystometry will reveal the filling phase as a low-pressure linear tracing. During this time, the patient will first perceive bladder filling at approximately 100-150 ml, but will continue to inhibit the bladder and will void only when filling sensation indicates approaching bladder capacity. During the filling phase, the bladder outlet remains closed with pressure always exceeding that in the bladder. Fluoroscopically, the bladder neck remains closed and totally leak-proof. Electromyographic recordings from the striated (external) sphincter show progressive recruitment of motor units throughout filling.

### MECHANISMS OF INCONTINENCE

Abnormalities of vesicourethral function giving rise to incontinence may be suitably classified in terms of failure to store, failure to empty, or a combination of both.

### Failure to Store

#### *Bladder Factors*

*Detrusor Instability:* This is defined as a detrusor contraction occurring during the filling phase of the micturition cycle. This term is used where the patient has no overt neurologic abnormality distinguishing this phenomenon from detrusor hyperreflexia.

Detrusor instability occurs in approximately 75% of patients with benign prostatic hypertrophy (Figure 2).[10] It is responsible for the irritative symptoms associated with this entity, namely frequency, urgency, and nocturia. If the amplitude of the unstable contraction is high, this may give rise to urge incontinence. Similarly, detrusor instability may occur without obstruction. Some evidence exists to suggest that this may be related to ischemia such that patients with degenerative vascular pathology may be more susceptible to this problem.

The reasons for this abnormal contractility remain unclear. Studies on both humans and animals with obstructive and idiopathic detrusor instability have shown that detrusor smooth muscle develops postjunctional supersensitivity that is characterized by reduced sensitivity to electrical stimulation and by greater sensitivity to acetylcholine, possibly due to partial parasympathetic denervation.[11] Detrusor smooth muscle in the idiopathic instability has a greater degree of spontaneous contractile activity and is more sensitive to lower frequencies of electrical activity or acetylcholine.[12]

Vasoactive intestinal polypeptide (VIP) has been shown to reduce spontaneous detrusor contractile activity and a reduction in the number of VIP interactive nerves and the VIP concentration has been found in biopsies of patients suffering from idiopathic detrusor instability.[13] Also, VIP reduces spontaneous detrusor contractile activity, which, as described, is successive in idiopathic detrusor instability. Further studies have shown a reduced cholinergic receptor and an increased alpha-adrenergic receptor density in the unstable detrusor. The significance of this observation is unknown, but may be evidence of reduced innervation. Ongoing studies will further define the abnormality of detrusor instability, ideally allowing more specific and effective therapies to be developed.[14]

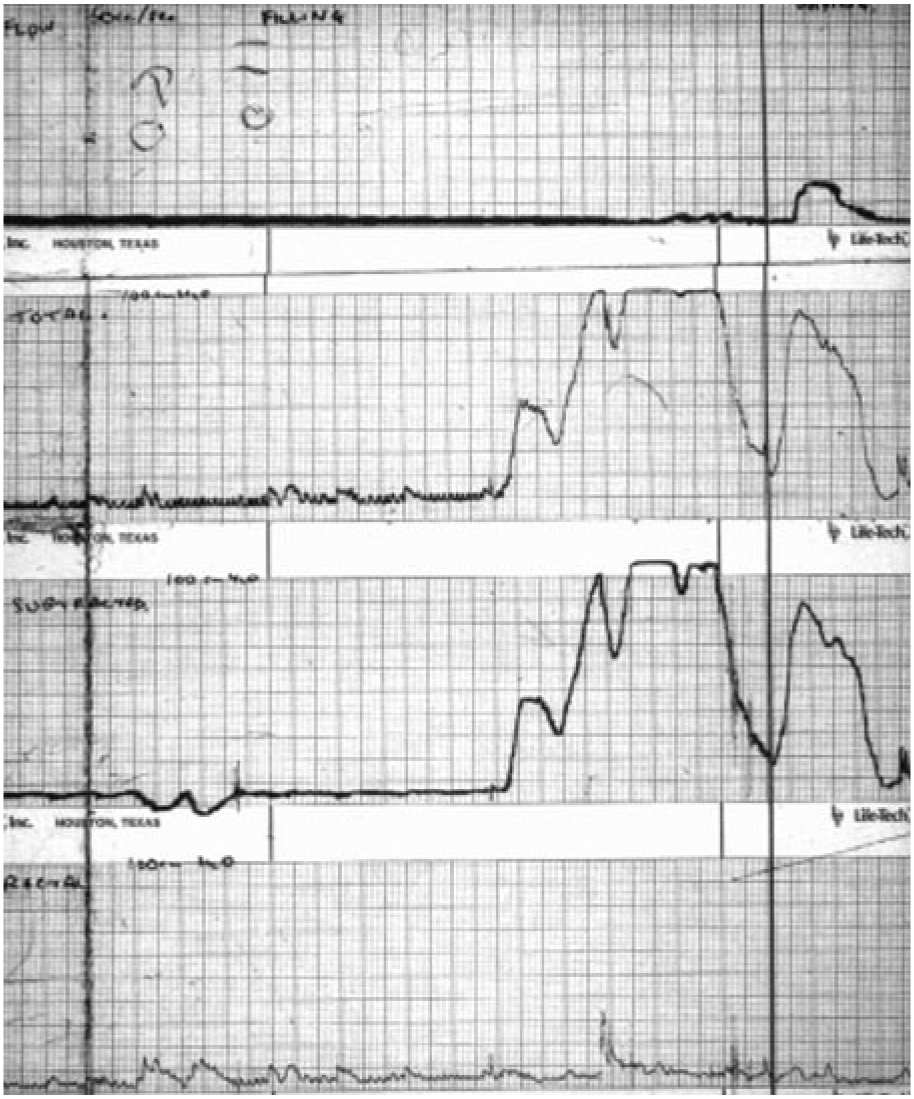

**FIGURE 2.** Sixty-one year old man with severe urgency, frequency, nocturia, occasional urge incontinence, and poor stream. Note high-pressure unstable contractions before voiding (>100 cm H2O). Patient voids with high detrusor pressure and low flow rate.

In approximately 10% of patients, this instability does not resolve following relief of obstruction. This phenomenon may occur because the instability arose *de novo* and was not related to obstruction or because the patient had some preexisting occult neuropathy. In these situations, the abnormal bladder contractility may give rise to urge incontinence.

*Detrusor Hyperreflexia:* Detrusor overactivity giving rise to urge incontinence may be neuropathic in origin. Hyperreflexia invariable accompanies spinal cord injury. This phenomenon may similarly be encountered in other spinal pathologies such as prolapsed intravertebral disks or degenerative spine disease. Cerebral vascular accidents, Parkinson's disease, and multiple sclerosis are often accompanied by this phenomenon. Diabetics do not always exhibit a sensory acontractile bladder and may behave in a hyperreflexic manner. This behavior will also give rise to urgency and urge incontinence. In some patients who undergo prostatectomy, surgery only exacerbates this primary problem. When confronted with a patient with symptoms of bladder outlet obstruction and a suggestion of a neuropathic element, multifunction urodynamic studies are mandatory in an attempt to identify remediable outlet obstruction.

*Low Compliance:* Inability to accommodate increasing volumes of urine may be due to anatomic or neuropathic factors. Anatomic factors affecting the viscoelastic properties of the bladder are seen in contracted bladder states. In its mildest form, the hypertrophied detrusor secondary to benign prostatic hypertrophy will exhibit mild to moderate impairment of compliance due to the increased rigidity of the thickened bladder wall. In more severe cases the detrusor is replaced by fibrosis. Irradiation, tuberculosis infection, multiple surgical interventions, or multiple resections may all cause this problem.

The most common neuropathic cause of poor compliance in the male patient is pelvic plexus injury. Although it would be expected that this lesion would cause an acontractile, low-pressure bladder, in reality it produces an acontractile, low-compliance bladder (Figure 3). This may be seen due to traumatic causes, namely pelvic fracture, but it is most common in patients who have undergone low anterior or abdominoperineal resection of the colon and rectum. Patients will often present with voiding dysfunction following these procedures and prostatectomy should only be contemplated if the bladder remains contractile and outlet obstruction can be definitely confirmed urodynamically. The causes of incontinence in this situation are complex. The abnormal bladder pressures may overcome outlet resistance, which in itself may be compromised neurologically or by previous prostatectomy. The acontractility will exacerbate the situation by causing inefficient emptying with overflow.

In a series of 215 men with post-prostatectomy incontinence, Leach et al found 15% had bladder dysfunction as the sole cause of their incontinence and 57% had bladder dysfunction as a major component.[15] Chao et al found 43% of post-prostatectomy patients had detrusor instability or decreased compliance, but this was the sole cause of incontinence in only 4%[16]. In our experience, in patients with significant incontinence undergoing artificial sphincter placement, abnormal detrusor characteristics were found in over 50% of cases. However, these were never the sole cause of leakage and intrinsic sphincteric weakness was found in all cases in addition to the detrusor abnormality. The presence of detrusor instability should always be confirmed and treated, but should not preclude management of the sphincteric weakness.

### Urethral Factors

The male sphincter mechanism is generally able to withstand the pressures placed upon it in daily life. Urethral incompetence is generally seen following prostatectomy. The incidence of post-prostatectomy incontinence differs significantly among studies depending on the definition of incontinence used. Many studies also combine urge and stress incontinence, thus the true incidence of sphincter weakness incontinence following prostatectomy is difficult to judge.

The incidence of incontinence following transurethral resection of the prostate or simple open prostatectomy ranges from 1% to 5%. The reported incontinence rate after radical prostatectomy has a much wider range. A review of the Johns Hopkins experience reports 92% of patients being totally dry, 6% wearing pads and only 0.3% requiring artificial urinary sphincter. Jønler et al used a questionnaire to assess leakage and bother. They reported 59% of the patients leaked daily, with 47% requiring pad use at some time. 30% leaked more than a few drops a day and 34% of the patients felt it was bothersome. Figures ranging from 3% to 75% have been reported in the past depending on the definition of incontinence used.[17]

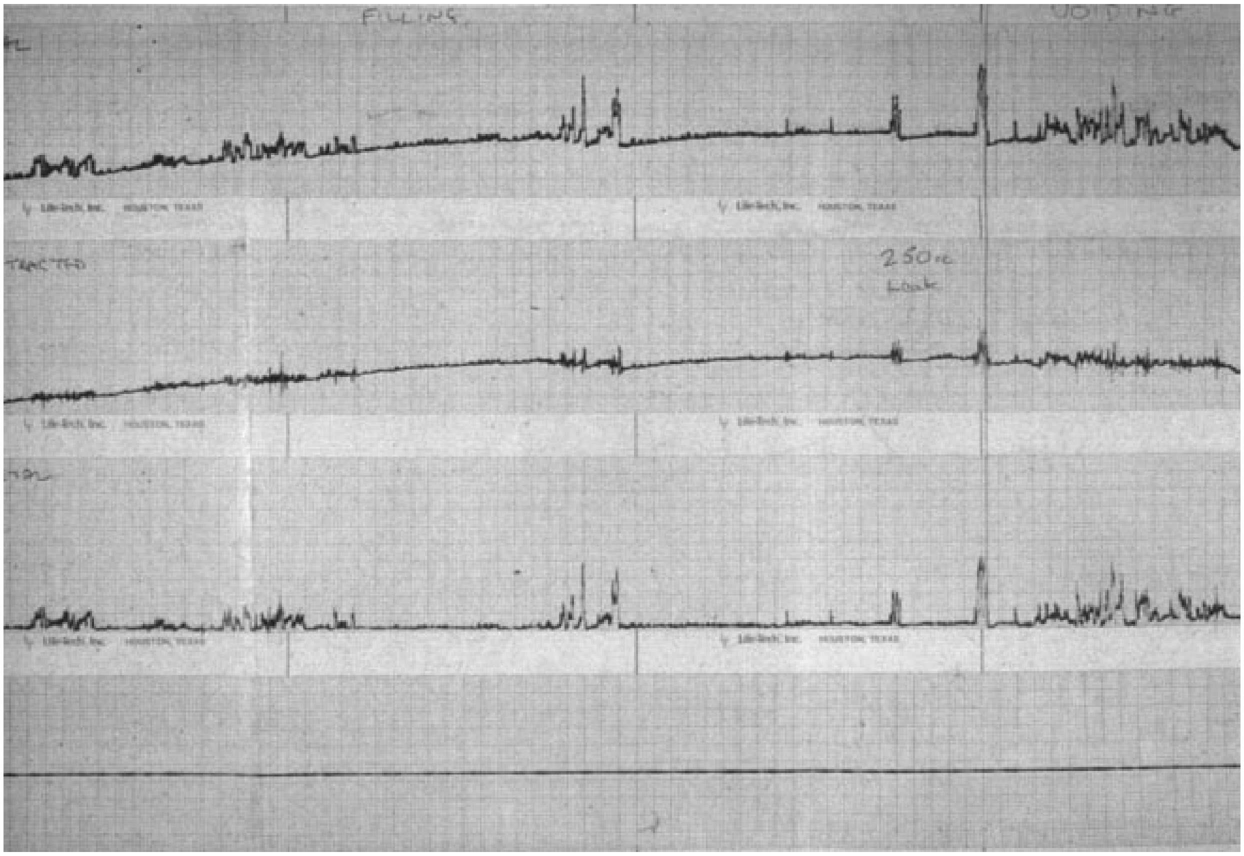

**FIGURE 3.** Forty-seven year old man with urinary incontinence; he had a history of low anterior resection of the colon and subsequent urinary incontinence. Incontinence worsened after TUR of prostate. Note steep filling cystometrogram (end-filling pressure of 200 mL, 60 cm H2O). Poor flow and intermittent pattern suggesting abdominal strain to empty.

Following any form of prostatectomy, maintenance of continence depends on the integrity of the distal urethral sphincter (intrinsic portion of the external sphincter). Preserving the bladder neck at simple open or transurethral prostatectomy or reconstructing the bladder neck at prostatectomy is not thought to influence continence significantly. Obviously, if the bladder neck ring can be totally preserved at radical prostatectomy, this will retain some degree of continence; however, in general, meticulous closure of the bladder neck around the catheter has no influence on postoperative continence.[18] Similarly, Licht et al found that preservation of the bladder neck did not have any impact on urinary control.[19] If the bladder neck can be preserved, the incidence of vesical neck contracture is lessened.

Improved continence rates following radical prostatectomy have been associated with the nerve sparing technique. O'Donell and Finan found their stress incontinence rate increased from 6% to 18% when comparing nerve-sparing versus non-nerve-sparing radical retropubic prostatectomy patients.[20] In contrast, we have not found any difference in the continence rates of these two groups. Of specific interest, post-prostatectomy incontinence in this study was most often seen in association with four specific features, namely increasing age, >1500 cc operative blood loss, post-op irradiation and anastomotic contracture.[21]

The functional length and position of the distal sphincter mechanism varies, as discussed previously. Although in general it corresponds to the area just distal to the verumontanum seen to constrict at endoscopy, it may vary considerable in length and occasionally extend proximally within the apex of the prostate. Myers has reported on two studies to support this thesis, one using cadaveric dissections and the other using retrograde urethrography. Both confirm the variability in length of the external striated sphincter, ranging from 1.2 to 5.0 cm.[22] Thus, those patients with shorter sphincters may be more at risk for post operative sphincteric incompetence.

A recent study by Bartsch et al using transurethral ultrasound has shown that post-prostatectomy incontinence patients differ from continent patients in several ways. They demonstrated either scaring, thinning, complete atrophy or minimal contractions in the rabdosphincter of 8 postprostatectomy incontinent patients compared to 40 continent subjects.[23]

Incontinence may occur after prostatectomy by damage to the sphincter at the time of surgery; alternatively, if the sphincter has been damaged prior to surgery, its incompetence may be unmasked by subsequent prostatectomy. In the first instance, sphincteric damage may occur by inadvertent resection distal to the verumontanum or by surgical excision during the distal dissection at radical prostatectomy. Unmasking of distal sphincteric incompetence usually occurs in relation to bulbomembranous stricture disease. Previous pelvic fracture and urethral disruption will destroy the distal mechanism. Similarly, strictures involving the bulbomembranous urethra treated by direct vision internal urethrotomy or open repair may compromise this mechanism. Patients requiring prostatectomy in these circumstances should be carefully counseled concerning the possibility of postoperative incontinence or should be managed by alternative measures.

### Failure to Empty

### *Bladder Factors*

Incontinence due to failure to empty the bladder is usually due to detrusor acontractility. Overstretching and decompensation of detrusor is commonly found in these situations and is usually seen in patients with chronic retention. Many such patients may recover some contractility with time, but a significant number will not empty despite prostatectomy. As discussed previously, diabetic patients with peripheral neuropathy may overstretch the bladder, giving rise to similar problems. The patient with a pelvic plexus injury, often after low colon or rectal excision, will develop a similar acontractile state. These patients will empty inefficiently and typically complain of overflow incontinence. They may be further complicated by a bladder exhibiting low compliance and sphincter weakness due to damage to the sphincter innervation during the bowel resection.

### *Urethral Factors*

Incomplete resection and/or stricture may compromise emptying, giving rise to overflow incontinence. This is a rare cause of incontinence following prostatectomy and detrusor factors should be excluded before making a secure diagnosis of residual obstruction. In any event, the patient with incontinence after prostatectomy should receive a comprehensive urodynamic work up before considering further resection.

### EVALUATION OF THE INCONTINENT MALE

Initial evaluation should include an accurate history emphasizing the nature of the incontinence, previous surgical procedures and concurrent illnesses and medications. Physical exam will include evaluation of mental status, assessment of neurological factors, palpation for bladder fullness and a complete urological exam.

Cystourethroscopy should be done to identify obvious sphincteric damage and exclude urethral stricture or bladder neck contracture. Although the stricture may not be the cause of the incontinence, it may need to be dealt with prior to any further management. The bladder should be examined to exclude stone, diverticulum, or tumor which might be exacerbating the problem or will need to be managed concurrently. Urine culture is always obtained and an upper tract assessment carried out by IVP or ultrasound if surgery is contemplated or a neuropathic etiology suspected.

In most cases of male incontinence, urodynamics should be done to accurately diagnose the problem. The specific indications for urodynamics are: previous prostatic surgery (however remote), neurologic history including diabetes, previous pelvic surgery (e.g. abdominoperineal resection of the colon) or symptoms and history that do not correlate.

As male incontinence is relatively rare and often complex, multifunction studies are recommended. Filling cystometry with provocative maneuvers to simulate incontinence episodes and a voiding study should be carried out. For this reason alone, water cystometry is preferred over $CO_2$ or air studies. The addition of fluoroscopy is especially helpful, as small amounts of leakage can be detected along with the detrusor event that precipitates it. It also allows visualization of the bladder outlet during storage and voiding.

If a neurologic etiology is suspected, it may be helpful to obtain an MRI of the lumbosacral spine and/or a neurologic opinion.

### MANAGEMENT

Management of the incontinent male patient depends on accurate diagnosis as outlined above. Most patients encountered will have outlet incompetence due to urethral sphincter damage. Detrusor factors contributing to incontinence are common, although rarely the sole cause. These factors must be identified or excluded so that a rational management plan can be arranged.

### Non-Surgical Therapy

#### *Behavioral Therapy*

Behavioral measures such as voiding chart, bladder training and pelvic floor exercises are useful after any prostatic surgery. They will help to re-establish normal voiding and improve minor degrees of incontinence, of both urge and stress types. In cases of true intrinsic sphincteric injury they are rarely helpful in the long term.

#### *Pharmacologic Therapy*

Alpha-adrenergic agents (e.g. ephedrine, pseudoephedrine, phenylpropanolamine) have the potential to improve outlet resistance because of the abundant alpha-adrenergic innervation in the bladder neck area. In practice, however, they are effective only for very mild degrees of leakage. It is difficult to assess in these circumstances whether there is any objective improvement in outlet resistance.

Anticholinergic therapy is routinely used empirically in the early treatment of post prostatectomy incontinence. As stated previously if detrusor instability is identified on urodynamic evaluation, anticholinergic therapy should be started with a view to controlling that these storage factors.

Ditropan (oxybutinin chloride), Levsin ( hyoscyamine), probanthine are several commonly used agents. They all vary in efficacy and have varying degrees of anticholinergic side effects (dry mouth, blurred vision, constipation). The tricyclic antidepressant Imipramine is a useful alternative. It is used in relatively small doses 75-100mg/day and is theoretically helpful with both detrusor instability and low compliance.

### Electrical Stimulation

Treatment similar to that used for female stress incontinence has been applied to the male patient. Anal plug electrodes have been used with only a 45% success rate. Length of follow-up in these cases is not recorded.[24] Sacral root and pudendal nerve stimulation has also been used for post-prostatectomy incontinence with only a 50% success rate.[25]

### External Appliances

External appliances such as penile clamp, condom appliance, diapers, and indwelling catheter are usually considered less socially acceptable. They are occasionally contemplated for minor degrees of leakage or in the patient with multiple other medical problems in whom a surgical option is deemed inappropriate.

### Surgical Therapy

### Urethral Injections - "Bulking Agents"

Periurethral or transurethral injection of substances to restore outlet resistance or urethral coaptation have been used for several years. The most utilized agents are Teflon paste, GAX collagen, autologous fat and silicon macroparticles. Experience with the two most popular agents is discussed.

*Teflon Paste:* Polytetrafluoroethylene (Teflon) paste injection was one of the first attempts to use urethral bulking agents for male incontinence . Politano and associates have been the major proponents of this method.[26] In the male, the paste is usually injected in the area of the distal mechanism by direct periurethral access via the cystoscope. Visible occlusion of the urethral lumen should be seen as the paste is injected submucosally. Problems have arisen due to migration of the material and it has been found in various sites some time after successful injection. If the urethra is scarred for any reason, this occlusion is difficult to obtain, rendering the procedure useless.

Although Politano's group was enthusiastic about this method due to its success and relative simplicity and have achieved 84% improvement in their series, other authors have not been able to reproduce these results. Due to difficulty with injecting Teflon, concerns of distant migration and potential difficulty with subsequent operations, Teflon injection is not regarded as a suitable option at this time.

*GAX Collagen:* Glutaraldehyde-treated bovine collagen has supplanted Teflon in more recent trials. GAX collagen induces only a minimal inflammatory response and has not been shown to migrate.[27] This material is similarly introduced suburethrally via a cystoscope. Published studies show that the technique is safe and may be repeated several times. Recent reports have shown increased success but do demonstrate that repeat injections are often required to achieve improvement in continence.

Aboseif et al reported on 88 patients with a 10 month follow-up.[28] 48% of patients were dry or nearly completely dry and another 38% were improved. Their patients required a mean of 3.5 injections and 25 ml of collagen. Patients required up to five injections, with some patients not showing improvement until after their fourth injection. 83% of patients had their injection under local anesthesia. Concomitant detrusor abnormalities were associated with a poorer response.

One of the disadvantages of collagen injection is the need for multiple injections. A possible explanation for this is that the transurethrally injected collagen is placedclose to the relatively non-compliant vesicourethral anastomosis. Klutke et al proposed injecting the collagen into the bladder neck area through an antegrade transvesical route.[29] Their technique requires dilation of a suprapubic cystostomy tract, with the collagen injected at several sites around the bladder neck through a cystoscope positioned via this access. Coaptation is visualized by a flexible cystoscope placed in standard fashion through the urethra. In their series, all patients had a single procedure with a mean injection volume of 14.5 ml. of collagen. They report on 20 patients at 8.5 months follow-up showing 45% significant improvement and a 25% cure rate.

Detrusor instability or hyperreflexia should be treated medically before any injection therapy is attempted. The University of Southern California group reported cure or improvement in 65% of their 46 patients.[30] Interestingly, only 21% of patients with detrusor instability or hyperreflexia had improvement, while 91% of those with stable bladders benefited.

### Artificial Urinary Sphincter

Three types of sphincter prostheses have been developed over the last 15-20 years. The Kaufman device provides constant compression of the bulbous urethra which can be adjusted percutaneously.[31] The Rosen prosthesis also incorporates a perineal compression balloon that can be deflated during voiding.[32] Both these devices have been superseded by the Brantley-Scott artificial urinary sphincter, which has been in development and use since 1972. The initial AS-721 sphincter used a compressive urethral cuff and pressure reservoir and had separate pumps for both inflation and deflation (Figure 4).

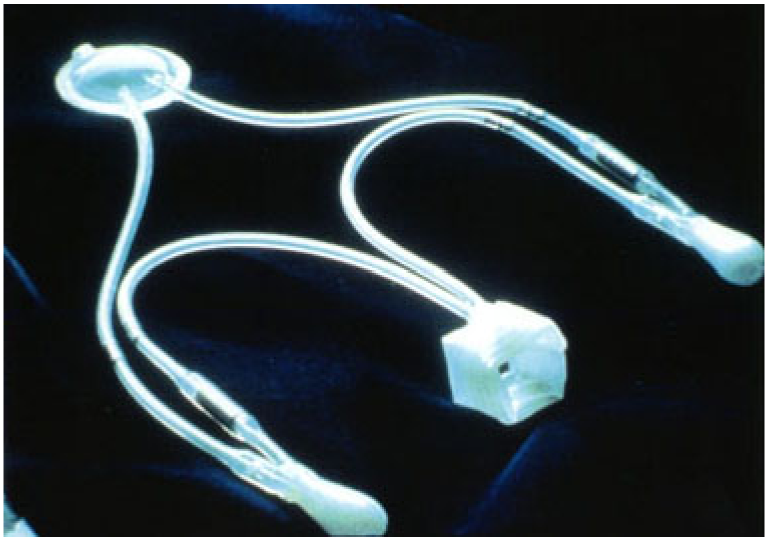

**FIGURE 4**. Brantley-Scott AS-721 artificial urinary sphincter.

The Scott sphincter in its present AS-800 form has several advantages over its predecessors (Figure 5). Increments of maximum compressive pressure are available, depending on the pressure reservoir implanted. The occlusive cuff may be placed around the bladder neck or bulbous urethra. The pump mechanism has a deactivation button that allows initial deactivation to prevent erosion and ease of activation obviates the need for additional surgery. Mechanical failure will not increase pressure against

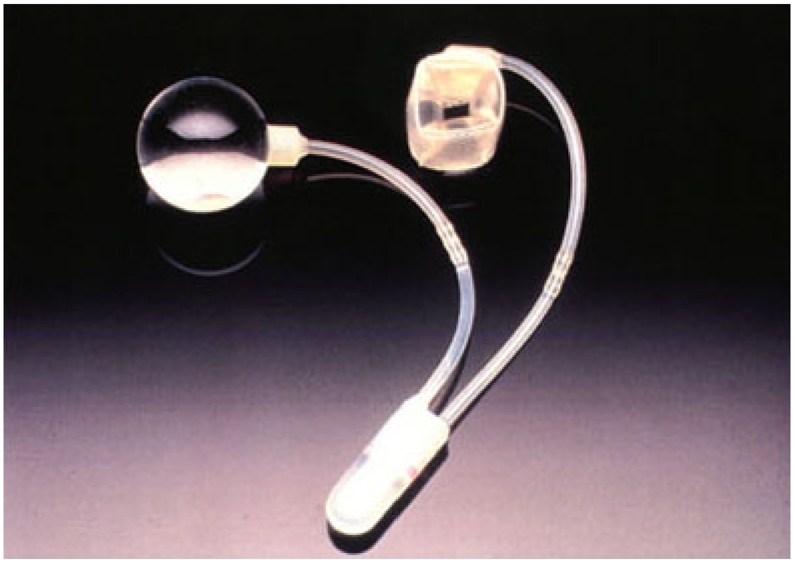

**FIGURE 5.** AS-800 artificial urinary sphincter.

the urethra in excess of that generated by the balloon. Technical refinements and the overall reliability have improved such that it now has a major role in the attainment of continence in a specific group of patients with urethral incompetence.[33]

The patient's suitability for this device should be ascertained by prior urodynamic evaluation, as many patients with post-prostatectomy incontinence will have associated abnormal bladder storage which should be treated appropriately before contemplating implantation. Endoscopy should have excluded strictures or fistulae and the urine should be sterile.[34]

The sphincter cuff is placed around the proximal bulb via a midline perineal incision. Initially, it was suggested that this be placed outside the bulbospongiosis muscle; however, this often led to recurrent incontinence due to atrophy of the tissue within the cuff. The present dissection includes separating this muscle off the bulbous urethra, which facilitates the development of a plane between the corpora and the proximal bulb. In most cases, a 4.5 cm occlusive cuff is used, but occasionally a 5.0 or 5.5 cm cuff is necessary. A 4.0cm cuff is now available and may be used when the urethra is naturally narrow. We do not advocate its use routinely, as the 4.5cm cuff works well in most cases and it is useful to reserve the smaller cuff in cases of urethral atrophy.

Once the cuff has been placed, the tubing from the cuff is transferred to a small suprapubic incision, generally made through the previous prostatectomy incision. The pressure reservoir can be placed intraperitoneally through the deep ring, but we find it more acceptable to split the rectus fascia in the midline and develop a subrectus pouch. Routinely, a 61-70 cm reservoir is used. If the patient has evidence of radiation damage or severe vascular compromise, a 51-60 cm balloon may be used. The amount of stress incontinence with this lower pressure reservoir is significant, so that it is only used in these compromised situations.

The pump mechanism is placed in a dependent superficial position in the scrotum via the suprapubic incision. The sphincter mechanism is filled with isotonic contrast medium to prevent fluid loss through osmotic transfer across the silastic material, and to allow radiologic identification of any subsequent leaks. The mechanism is left deactivated for at least six weeks to prevent erosion. At this time, the sphincter is easily activated in the office and the patient is taught to use the device.

In patients with urethral sphincter weakness due to a neurological etiology or after posterior urethral disruption, the cuff should be placed around the bladder neck. This is achieved through a midline or Pfannenstiel incision. The retropubic space is entered and a plane is developed around the bladder neck and proximal urethra. This dissection is often facilitated by opening the bladder. A larger cuff is used in these situations, ranging in size from 6 to 11 cm depending on the dissection and the patient's size. The reservoir is placed lateral to the bladder below the peritoneum.

A 71-80 cm reservoir is routinely used, assuming the cuff size is 6.5 cm or greater and that the dissection has not compromised the bladder neck or proximal urethra in any way. If this situation should occur, the procedure should be abandoned or the surgeon should consider repairing the bladder neck with an omental wrap to prevent subsequent cuff erosion. Some authors recommend a more distal placement of the cuff in this situation, incising the pubourethral sling, and developing the natural plane between the apex of the prostate and pubourethral sling. The mechanism is again deactivated for at least six weeks.[35]

Satisfaction rates for the artificial urinary sphincter are high. We reviewed our experience over the last nine years to assess continence and patient satisfaction. An impartial reviewer used a telephone questionnaire to gather data. 92% of patients were continent or improved and 96% said they have or would recommend the artificial urinary sphincter to a friend. Relative improvement in continence was the most significant factor affecting patient perceived outcome.[36]

### Incontinence Following Artificial Sphincter Placement

### *Patient-Related Problems*

The most common cause of incontinence following activation of an artificial urinary sphincter is the patient's inability to empty the cuff correctly and subsequent overflow incontinence. This problem can be avoided by carefully counseling the patient before surgery.

### *Fluid Loss*

Fluid loss due to disruption of a connection or a pinhole leak, usually in the cuff, can occur at any time following activation--up to several years after implantation. This is diagnosed by loss of contrast seen radiologically within the mechanism, associated with poor refilling of the pump mechanism. This problem must be corrected surgically. The sphincter connections are approached through the suprapubic wound. If these are still intact, irrigation of the cuff will usually identify the leak (Figure 6). At this time, the cuff should be replaced either perineally or via the suprapubic incision, depending on the original site of insertion. It is easy to replace because of the false capsule that forms around the cuff. If the leak has been present for some time, it is advisable to replace the whole sphincter, as minute particles of debris within the system may cause subsequent pump malfunction.

### *Erosion*

Cuff erosion is fortunately rare now that delayed activation is common practice.[37] Most cases of erosion seen today are related to inappropriate urethral instrumentation, often without deactivation of the cuff.[38] Erosion is also seen occasionally in patients who have suffered pelvic fracture, when the bladder neck area has been compromised by the trauma. Erosion is characterized by pain, hematuria, dysuria, and recurrent incontinence. It is diagnosed endoscopically. If encountered early, it may be managed by removal of the cuff only. Generally, the whole prosthesis may be a source of infection and thus all three parts should be removed. Occasionally the erosion is large enough to require urethral repair. Reimplantation should not be considered for six months, and strictures at the erosion site should be dealt with before considering this procedure.

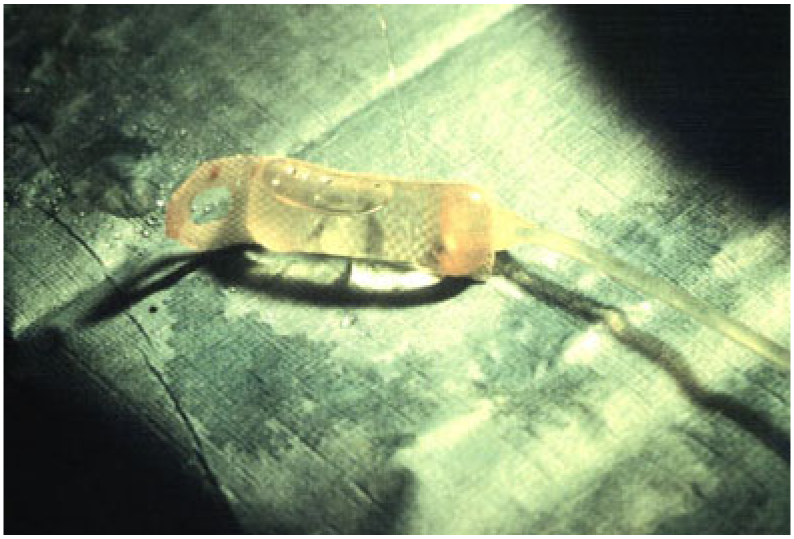

**FIGURE 6**. Pinhole leak from occlusive cuff of artificial urinary sphincter removed at surgical revision.

### Bladder Factors

Abnormal bladder storage may compromise the results of artificial urinary sphincter implantation. Detrusor instability or poor compliance may have been present at the time of implantation or may arise *de novo* once the bladder can store urine after implantation. Abnormal detrusor function should always be identified before implantation and treated appropriately by pharmacologic means. Despite this precaution, this management may be inadequate after sphincter activation.

### Urethral Factors

Occasionally the bulbomembranous urethra is so scarred that appropriate coaptation of the urethra is prevented. More commonly, after a variable period of time, the urethra within the cuff may atrophy, reducing the devices ability to coapt. The phenomenon of urethral atrophy is suspected where the sphincter mechanism appears to be functioning appropriately, but leakage slowly increases at any time between 6 months to several years after implantation. The patient may describe having to increase the number of pumps to empty the cuff, corresponding to the increased amount of fluid allowed into the cuff. This problem may be managed in several ways. The simplest method is to exchange the existing cuff to the next smallest size. Prior to the availability of the 4.0cm cuff, we successfully managed this problem by repositioning the cuff in a more proximal position around the urethra. The proximal edge of the existing false capsule is used to 'fix' the cuff in its new proximal position. Another useful technique in this situation is to implant double cuffs. A 'Y' connector allows this technique to be used with a single pump and reservoir.

### Urethral Slings

The sling procedure has gained increasing popularity in the management of female incontinence due to urethral sphincter weakness.[39] The first use of this technique was in post-prostatectomy patients,[40] a rectus fascial sling was used in this situation. Recently, this technique has been revived as an alternative to artificial urinary sphincter with good results claimed in a small group of neurogenic patients. It remains to be seen whether this technique will be suitable for post-prostatectomy incontinence.[41]

This procedure is approached in a similar fashion to bladder neck sphincter placement. The retropubic space is entered and a plane dissected around the proximal urethra and bladder neck. The sling is usually taken from the rectus fascia approximately 1.5 cm wide with the central portion cut approximately 2 cm wide. The lower end of the sling in the midline approach or one side of that taken by a Pfannenstiel incision may be left attached, or a shorter fascial strip may be used suspended by prolene sutures.. The sling is then passed around the previously dissected plane and sutured to the rectus fascia on either side with several non-absorbable sutures. The sling should be tightened sufficiently to coapt the proximal urethra.

If the bladder neck is wide open, it is debatable whether a sling in appropriate; however, this problem may be managed by V-Y reconstruction of the bladder neck. In these cases, the bladder should be drained by suprapubic catheter rather than urethral Foley.

At the present time standard surgical treatment of post prostatectomy incontinence rests with either artificial sphincter or collagen injection. In most cases the sphincter works well and is well accepted by the patients. The results of collagen injection are less reliable although its success may be improved by the antegrade technique described.

### Management of the Devastated Urethra

After severe urethral trauma, often after pelvic fracture, or in patients who have undergone multiple surgical procedures, the urethra may be unreconstructable and a source of intractible incontinence. Long transphincteric strictures and urethrocutaneous fistulae may be associated features in this situation. In the past, this patient would have been managed by indwelling urethral or suprapubic catheter or would have undergone conduit supravesical diversion. At present, suitable rehabilitation of the lower urinary tract can be achieved by bladder neck closure and construction of a continent catheterizable abdominal stoma.

### Bladder Neck/Urethral Closure

In the male this may be approached perineally or suprapubically. We favor the suprapubic approach especially in neurologically compromised patients. Although perineal urethral closure is easier, the risk of fistula formation is higher. In addition suprapubic bladder neck closure fasclitates interposition of omentum or rectus muscle to aid healing, and appropriate bladder drainage procedures can be carried out through the same incision.

In this technique, the bladder neck and proximal urethra are opened anteriorly to facilitate separation of the posterior bladder neck from the urethra.

In these situations, bladder emptying may be accomplished by placement of a supra-pubic catheter or it may be deemed appropriate to construct a continent catheterizable stoma. Several techniques are available to accomplish this, namely the Kock intussuscepted ileal nipple, a Benchekroun-type nipple[42] and the Mitrofanoff procedure (Figure 7). In general the latter is the easiest to construct and the most reliable long term. In most cases the appendix is utilized but a suitable narrow tube can be constructed from any segment of bowel.[43]

### CONCLUSION

Male incontinence remains a challenge for the urologist, but as we have described, careful diagnostic steps allow the use of increasingly effective treatment modalities. Better methods of improving abnormal bladder storage and simpler techniques of enhancing outlet resistance will be helpful to the patient. Attention to detail will prevent this problem in many patients. Ideally, a better understanding of the mechanisms of continence and the nature of the sphincter mechanism will prevent subsequent incontinence.

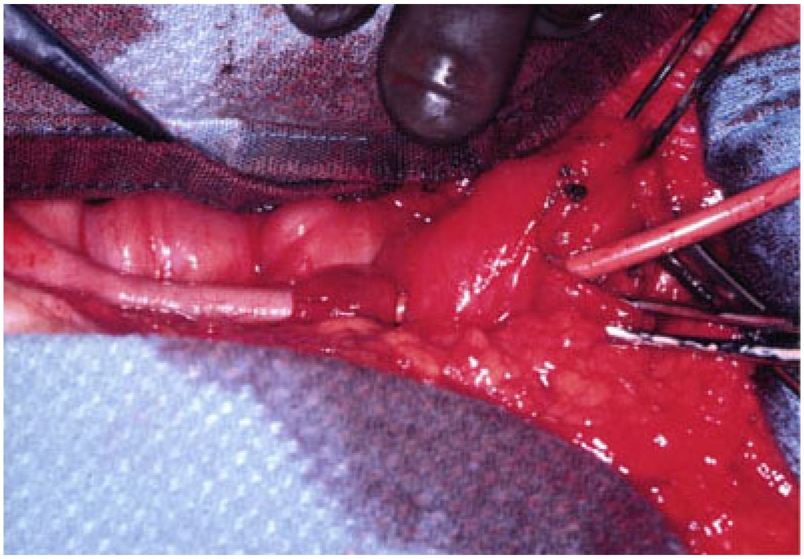

**FIGURE 7**. Mitofanoff catheterizable stoma: appendix implanted into augmented bladder to provide continent catheterizable access to bladder.

### REFERENCES

1. Turner-Warwick R. Observations on the function and dysfunction of the sphincter and detrusor mechanisms. Urol Clin N Amer 1979; 6:113.
2. Caine N, Raz S, Ziegler M. Adrenergic and cholinergic receptors in the human prostate, prostatic capsule, and bladder neck. Br J Urol 1975; 47:93.
3. Tanagho EA. Anatomy of the lower urinary tract. In: Walch PC, Gittes RF, Perlmutter AD et al, eds. Campbell's Urology, 5th ed. Vol 1. Philadelphia: WB Saunders, 1986, p.46. (Check references/update).
4. Turner-Warwick R, Whiteside CG, Worth PHL, Mueller EJG, Bates CP. Urodynamic review of clinical problems associated with bladder neck dysfunction and treatment by endoscopic incision, transtrigonal posterior prostatectomy. Br J Urol 1973; 45:44.
5. Turner-Warwick R. The relationship of prostatic enlargement to the distal sphincter mechanism, the bladder neck mechanism, dyssynergic bladder neck obstruction. In: Hinman F, Chisolm B, eds. Benign Prostatic Hypertrophy. New York. Spring-Verlag, 1983.
6. Light JK. Continence mechanisms following orthotopic bladder substitution. In: King LR, Stone AR, Webster GD, eds. Bladder Reconstruction and Continent Urinary Diversion, 2nd ed. St. Louis: Mosby Yearbook, 1991, p.441.
7. Chilton CP. Distal urethral sphincter mechanism in the pelvic floor. In: Mundy AR, Stephenson TP, Wein AJ, eds. Urodynamics. London: Churchill-Livingston, 1984, p. 9.
8. Gosling JA, Dixon IS, Critchley HOD, Thompson SA. Comparative study of the urinary external sphincter and periurethral levator muscle. Br J Urol 1981; 53:35.
9. El Badawi A, Atta MA. Ultrastructure of vesicourethral innervation: evidence for somatomotor plus autonomic innervation of the male feline rhabdosphincter. Neurourol. Urodynam 1985; 4:23.
10. Abrams P. Detrusor instability and bladder outlet obstruction. Neurourol Urodynam 1985; 4:317.
11. Sibley GNA. Experimental model of detrusor instability in the obstructed pig. Br J Urol 1985; 50:72.
12. Kinder RB, Mundey AR. Pathophysiology of idiopathic detrusor instability and detrusor hyperreflexia: an in vitro study of human detrusor muscle. Br J Urol 1987; 60:509.
13. Gu J, Restorick JM, Black MA, et al. Vasoactive intestinal polypeptide in the normal and unstable bladder. Br J Urol 1983; 55:645.
14. Restorick JM, Mundy AR. The density of cholinergic and alpha- and beta-adrenergic receptors in the normal and hyperreflexic human detrusor. Br J Urol 1989; 63:32.
15. Leach GE, Trockman B. Wong A, Hamilton J, Haab F, Zimmern PE. Post-prostatectomy incontinence: urodynamic findings and treatment outcomes. J Urol 1996; 155:1256.
16. Chao R. Mayo ME: Incontinence after radical prostaetctomy: detrusor or sphincter causes. J Urol 1995; 154:16.
17. Foote J, Yun S, Leach GE. Post-prostatectomy incontinence. Urol Clin North Am 1991; 182:229.
18. Steiner SS, Morton RA, Walsh PC. Impact of anatomical radical prostatectomy on urinary incontinence. J Urol 1991; 145:512.

19. Licht M. R., Klein E. A., Tuason L, Levin H., Impact of bladder neck preservation during radical prostatectomy on continence and cancer control, Urology 1994, 44, 6: 883-885
20. O'Donnell PD, Finan BF, Barnett T, Brookover T. Continence recovery following radical prostatectomy. Neurourol Urodynam 1990; 9:251.
21. Kim KB, Litwiller S, Stone AR. Can incontinence following radical prostatectomy be prevented? Br J Urol 1996; 77:51.
22. Myers RP. Male urethral sphincteric anatomy and radical prostatectomy. Urol Clin North Am 1991; 18:211.
23. Strasser H et al. Transurethral ultrasound: evaluation of anatomy and function of the rhabdosphincter of the male urethra. J Urol 1998; 159:100-105
24. Sotiropoulos A, Yeaw S, Lattimer JK. Management of urinary incontinence with electronic stimulation: observations and results. J Urol 1976; 116:747.
25. Schmidt RA. Applications of neurostimulation in urology. Neurourol Urodynam 1988; 7:585.
26. Politano VA, Small MP, Harper JM, Lynne CM. Periurethral Teflon injection for urinary incontinence. J Urol 1974; 111:180.
27. Shortliffe L.M.P, Freiha F.S, Kessler R, Stamey T.A, Constantinou C.E. Treatment of urinary incontinence by periurethral implantation of glutaraldehyde cross-linked collagen. J Urol 1989; 141:538.
28. Aboseif SR, O'Connell HE, Usui A, McGuire EJ. Collagen injection for intrinsic sphincteric deficiency in men. J Urol 1996; 155:10.
29. Klutke CG, Nadler RB, Tiemann D, Andriole GL. Early results with antegrade collagen injection for post-radical prostatectomy stress urinary incontinence. J Urol 1996; 156:1703.
30. Martins FE, Bennett CJ, Dunn M, Filho D, Keller T, Lieskovsky G. Adverse prognostic features of collagen injection therapy for urinary incontinence following radical retropubic prostatectomy. J Urol 1997; 158:1745.
31. Kaufman JJ. The silicon gel prosthesis for the treatment of male urinary incontinence. Urol Clin North Am 1979; 52:393.
32. Rosen M. A simple artificial implantable sphincter. B J Urol 1976; 48:675.
33. Scott FB, Brantley WE, Tim CW. Treatment of urinary incontinence by an implantable prosthetic sphincter. Urology 1973; 1:252.
34. Mundy AR, Stephenson TP. Selection of patients for implantation of the Brantley-Scott artificial urinary sphincter. Br J Urol 1984; 56:717.
35. Webster GD, Sihelnik SA. Troubleshooting the malfunctioning Scott artificial urinary sphincter. J Urol 1984; 131:269.
36. Litwiller SE, Kim KB, Fone PD, de Vere White RW, Stone AR, J. Urol.1996, 156, 1975-1980.
37. Goldwasser B. Avoiding the problems of artificial urinary sphincter implantation. Probl Urol 1990; 4:187.
38. Fishman JR, Stone AR. Artificial urinary sphincter erosion in cardiovascular surgical patients. West J Med 1991; 154:6728.
39. McGuire B, Litton B. Pubovaginal sling procedure for stress incontinence. J Urol 1978; 119:82.
40. Millin P. Retropubic urinary surgery. Baltimore: Williams & Wilkins, 1947: 12.
41. Raz S, McGuire EJ, Erlich RM. Fascial sling to correct male neurogenic sphincter incompetence: the McGuire/Raz procedure approach. J Urol 1988; 139:528.
42. Benchekroun A. The ileocecal continent bladder. In: King LR, Stone AR, Webster GD, ed. Bladder Reconstruction and Continent Urinary Diversion. 2nd ed. St. Louis: Mosby Yearbook, 1991:324.
43. Woodhouse C.R. The Mitrofanoff principle for continent urinary diversion. World J Urol 1996; 14:99.

**This article should be referenced as follows:**

Stone, A.R. and Nelson, R.S. (2004) Evaluation and management of male urinary incontinence. *TheScientificWorldJOURNAL* **4 (S1)**, 330–345.

**Handling Editor:**

Anthony Atala, Principle Editor for *Urology* — a domain of *TheScientificWorldJOURNAL.*
